# Oral Sodium Butyrate Is Associated with Altered Tissue Bacterial Antigens and Innate Immune Marker Expression Across Different Organs in Athymic Mice

**DOI:** 10.3390/nu18162604

**Published:** 2026-08-10

**Authors:** Abubakr H. Mossa, Walaa Sherif Mohammed Alhalabi, Sylia Chehade, Razaz Omer Osman Alnakhli, Ibrahim Yaseen Hachim, Mahmood Y. Hachim

**Affiliations:** 1 Research Institute for Medical and Health Sciences, University of Sharjah, Sharjah 27272, United Arab Emirates; 2 College of Medicine, University of Sharjah, Sharjah 27272, United Arab Emirates; 3 College of Medicine, Mohammed Bin Rashid University of Medicine and Health Sciences, Dubai 505055, United Arab Emirates

**Keywords:** butyrate, gut-lung-kidney axis, LPS, athymic mice

## Abstract

**Background:** Butyrate is a microbiota-derived short-chain fatty acid with established roles in maintaining intestinal barrier integrity and regulating immune responses. Although its effects have been extensively investigated in immunocompetent models, systemic immune homeostasis in severely immunodeficient hosts remains incompletely understood. This exploratory study evaluated the impact of sodium butyrate (NaB) supplementation on tissue-associated microbial antigen detection and immune marker distribution along different organs in athymic nude mice. **Methods:** Female NU/J athymic mice were administered either regular drinking water (H_2_O, *n* = 10) or NaB-supplemented water (150 mM, *n* = 5) for six weeks. Intestinal, liver, lung, and kidney tissues were collected for histopathological and immunohistochemical analyses. Expression of microbial antigens, including lipopolysaccharide (LPS) and lipoteichoic acid (LTA), as well as immune-associated markers (CD3, CD8a, CD68, CD278, and transglutaminase-2 [TGM2]), was assessed using a semiquantitative scoring system. **Results:** NaB supplementation did not significantly affect organ morphology but was associated with better weight gain. Histological examination revealed preserved tissue architecture in all organs, with only mild condensation of small intestinal crypts in NaB-treated mice. Immunohistochemical analysis showed consistently lower LPS expression in NaB-treated animals compared with controls in the small intestine, liver, and kidney, with a borderline reduction in the lungs. Reduced LPS detection was accompanied by significantly lower expression of the macrophage marker CD68 in the kidney. **Conclusions:** Oral NaB supplementation was associated with lower detection of bacterial antigens in tissues and reduced innate immune expression across multiple organs. These findings support a potential role for butyrate in limiting microbial expression and systemic inflammatory signaling in athymic mice.

## 1. Introduction

Butyrate, a major short-chain fatty acid (SCFA) produced by microbial fermentation of dietary fiber, is a key metabolite for intestinal health [1]. Its luminal presence favors butyrate-producing bacteria and supports epithelial barrier integrity by upregulating tight junction proteins, enhancing mucus production, and stabilizing hypoxia-inducible factor, thereby limiting microbial translocation [2]. In the intestine, butyrate is taken up predominantly in the colon via passive diffusion and active transport through Sodium-coupled Monocarboxylate Transporter 1 (SMCT1) and Monocarboxylate Transporter 1 (MCT1), whose expression is regulated by luminal SCFA availability and altered in dysbiosis or low-fiber states. As a signaling molecule, butyrate activates SCFA-sensing G protein-coupled receptors (GPCRs), such as GPR41, GPR43, and GPR109A, on epithelial cells, thereby inducing serotonin release and enhancing water and electrolyte absorption [2,3], while in immune cells, it initiates anti-inflammatory cascades that help maintain mucosal homeostasis [1].

In addition to its role in mucosal barrier integrity, butyrate acts as a potent inhibitor of histone deacetylases (HDACs), altering the accessibility of key immune- and barrier-related genes in intestinal epithelial and immune cells, including T cells, dendritic cells, and macrophages. In T lymphocytes, butyrate enhances acetylation at the Foxp3 locus, promoting the differentiation and stability of extrathymic regulatory T cells that constrain intestinal inflammation [4]. HDAC inhibition by butyrate can also reduce constitutive TNF-α-induced NF-κB activation in colonic cells and the downstream expression of proinflammatory genes [5]. Beyond its purely epigenetic actions, butyrate also signals through GPR43 and GPR109A on dendritic cells, macrophages, and T cells, skewing cytokine profiles toward an anti-inflammatory milieu characterized by reduced IL-12 and IFN-γ and enhanced IL-10 production [6,7]. Therefore, butyrate locally reprograms intestinal epithelial and immune cells via HDAC inhibition and receptor signaling, reducing NF-kB-mediated inflammation and promoting regulatory, tolerance-inducing cells, so disruptions in its production or transport are closely linked to intestinal inflammatory diseases [2,8].

Butyrate is also a key metabolite linking the intestine to pulmonary immunity through the gut–lung axis, as it can enter the circulation from the intestine and reach the bone marrow and lungs, where it influences hematopoiesis and local immune-cell function [9]. Circulating butyrate conditions bone marrow progenitors to generate fewer proinflammatory monocytes and dendritic cells, which subsequently seed the lung. The resultant reduced capacity to drive inflammation broadly dampens airway hyper-responsiveness. In experimental models of allergic asthma and viral or bacterial lung infection, increased intestinal butyrate—either via high-fiber diets, direct butyrate supplementation, or enrichment of butyrate-producing bacteria—has been associated with decreased eosinophilic infiltration, lower levels of cytokines such as IL-4, IL-5, IL-13, and IL-17, and improved airway remodeling, mucus production, and lung function indices [9,10,11]. These combined systemic and local effects are particularly significant in modulating the severity of respiratory infections by enhancing barrier integrity and controlling excessive immune activation in the lung.

The gut–kidney axis also benefits from butyrate in the intestine. Butyrate produced in the colon is distributed through circulation to the kidneys, where it acts via its receptors on renal and vascular cells [12]. In the context of kidney inflammation, butyrate exerts antioxidant, anti-inflammatory, anti-fibrotic, and anti-apoptotic effects across multiple experimental models, including septic kidney injury and diabetic kidney disease [12,13,14]. Mechanistically, butyrate attenuates oxidative stress. It preserves mitochondrial function, activates protective signaling, limits activation of proinflammatory pathways, including NF-κB and TLR2/4, reduces cytokines such as TNF-α and IL-6, and decreases tubular and glomerular cell death [12]. In an animal model of chronic kidney disease, butyrate administration improved intestinal tight junctions, reducing leakage of gut-derived toxins, such as bacterial lipopolysaccharide (LPS) [15]. These findings highlight the significance of this metabolite as a potential therapeutic target within the gut–kidney axis.

Beyond immunocompetent in vivo models, the role of butyrate in immune and multisystem integrity in immunodeficient animal models remains to be further validated. Through its epigenetic and receptor-mediated pathways, butyrate can reshape immune responses in immunocompromised mice. In cyclophosphamide-induced immunosuppressed mice, strategies that enrich butyrate-producing bacteria or increase colonic butyrate restore splenic and intestinal lymphocyte numbers, elevate secretory IgA, and normalize cytokine profiles, indicating a partial recovery of mucosal and systemic immune competence [16]. This can be beneficial in immunocompromised hosts where dysregulated inflammation coexists with defective pathogen control. In autoimmune-prone models, butyrate-prodrug supplementation can increase myeloid-derived suppressor cells and regulatory T cells, reduce NF-κB-driven cytokines such as TNF-α and IFN-γ, and constrain pathogenic infiltration into target organs [16,17]. Together, these data suggest that in immunocompromised mice, controlled elevation of butyrate or enhancement of butyrate-producing microbiota can improve barrier integrity and immune homeostasis. Still, its net effect depends on the underlying defect, disease context, and dose, emphasizing the need for tailored interventions in future preclinical and translational studies.

This pilot study aims to demonstrate, in exploratory settings, the benefits of butyrate supplementation on structural integrity and innate immune cell expression in the small intestine, liver, lung, and kidney in an immunodeficient athymic mouse model. The primary outcome of the study was to compare LPS expression in the small intestine of the two groups, the organ most directly exposed to NaB. Secondary outcomes include weight change, microbial and immune marker expression in the small intestine and other organs (including liver, lungs, and kidneys).

## 2. Materials and Methods

### 2.1. Animal Model and Ethical Statement

All procedures involving animals were carried out in accordance with the institutional guidelines for the care and use of laboratory animals and approved by the Institutional Animal Care and Use Committee at the University of Sharjah, Sharjah, UAE (no. ACUC-27-02-2024). For this pilot study, 15 female nude mice NU/J (HOM), aged 5–7 weeks, were used in all experiments. These mice were selected for their Foxn1 mutation, which causes a defective thymus. This results in hairlessness and severe immunodeficiency due to a lack of T cells. Animals were kept under specific pathogen-free (SPF) conditions in individually ventilated cages with controlled temperature (22 ± 2 °C), humidity (50 ± 10%), and a 12-h light/dark cycle. Standard chow and water were provided ad libitum, and mice were allowed to acclimate for one week before any experimental procedures.

### 2.2. Sodium Butyrate Treatment

The mice were randomly divided into two groups to examine the effect of sodium butyrate (NaB). No specific randomization method was used; cages with 2–5 mice were allocated by one researcher (AM) regardless of exact birth date or weight. Cages were coded with letters of the alphabet, and the tissue samples were coded accordingly. The histopathologist was blinded to the group allocation till the staining and scoring were completed. NaB (Aldrich, St. Louis, MO, USA; Cat. No. 303410) was added to the drinking water of one group at 150 mM 7 days after acclimatization, while the control group received regular drinking water. The drinking supply was replaced weekly with a fresh mixture. Other than checking the solution’s odor and turbidity, we did not take any measures to monitor changes in NaB concentration. Even if the solution looked fresh, the mixture was changed weekly. The general health and behavior of the mice were recorded daily to ensure animal welfare. Their weight was monitored weekly. No obvious differences in drinking behavior or hydration status were observed between groups. Researchers involved in mouse follow-up were not blinded to the treatment. Given the observed animal drinking habits and the established model, we did not measure NaB in the body tissues or fluids.

### 2.3. Tissue Collection and Processing

At the experimental endpoint (6 weeks), mice were anesthetized and euthanized by exsanguination. Body tissues, including liver, small intestine (mainly jejunal segments), kidneys, and lungs, were collected and divided into two specimens: one fixed in 10% neutral-buffered formalin for histopathological and immunohistochemical analyses, and the other snap-frozen in liquid nitrogen and stored at −80 °C for further studies.

### 2.4. Sample Processing and Embedding of Tissues

Mouse tissues were sectioned, preserved in 1X Phosphate-Buffered Saline (PBS), and transferred to formalin for further processing. Tissues were fixed in 10% neutral buffered formalin (Fisher Scientific, Waltham, MA, USA; Cat. No. 22050104) for 8 h at room temperature. The tissues placed in cassettes then processed with Leica biosystems 1020 tissue processor by passing through: container 1 and 2 10% neutral buffered formalin for fixation (1 h each), dehydration done through series of graded ethanol as 50%, 70%, 90%, 95%, 100% and 100% (for 45 min in each), clearing done with xylene1 and 2 (for 1 h in each), and impregnation done by putting the cassette in melted paraffin wax 1 and 2 (for 1:30 h each).

### 2.5. Formalin-Fixed Paraffin-Embedded (FFPE) Sectioning and Hematoxylin and Eosin Staining of Tissues

The processed tissues were embedded in paraffin (FFPE), and sections were cut at a thickness of 4 micrometers. These sections were then placed on a positively charged slide (Thermo Fisher Scientific, Waltham, MA, USA; Cat. No. MIC3040). Hematoxylin (Thermo Fisher Scientific, Waltham, MA, USA; product no. 6765003) and eosin (Thermo Fisher Scientific, Waltham, MA, USA; Cat. No. 22-050-198) staining was performed as follows. The process involved deparaffinization with xylene (xylene 1 & 2 for 10 min each), followed by rehydration through a series of graded ethanol solutions (100%, 90%, 80%, 70%, and 50%) for 2 min each, then transferred to distilled water. Hematoxylin staining was performed, followed by water washing, eosin counterstaining, dehydration, clearing, and mounting. Images that do not reflect the full architecture of the organ were excluded as follows: for the small intestine: full wall thickness; liver: at least four hepatic lobules; lung: the section should show at least one respiratory bronchiole with alveolar sacs; kidney: full thickness of the cortex. Samples from one H_2_O mouse were excluded for LTA, CD68, and TGM. If the section met the criteria, one section was used per animal, then 2 or more areas of interest (AOI) were used for evaluation.

### 2.6. Antigen Retrieval

Antigen retrieval buffer was prepared according to the antibody used: Sodium citrate buffer (pH 6.0) for CD278, CD8a, TGM2, and LTA, and EDTA buffer for CD3, CD68, and LPS. Slides were placed in their corresponding buffers and kept in a water bath at 95 °C for 45 min. Then they were kept at room temperature for 20 min to cool, washed in running tap water for 10 min, and then rinsed in distilled water. Tonsils were prepared as positive controls and stained as the mouse batch samples. Smears of Gram-negative and Gram-positive bacteria were fixed and used as positive controls for Anti-LPS and Anti-LTA, respectively. Refer to the supplementary figures for the positive control images (Table S4). Table 1 shows the details of the antibodies.

### 2.7. Slide Preparation and Staining

Immunohistochemistry was performed using the HRP/DAB Detection IHC Kit (Abcam, Cambridge, UK; Cat. No. ab64264) according to the manufacturer’s instructions. Tissue sections were first outlined with a PAP hydrophobic pen (Abcam, Cambridge, UK; Cat. No. ab2601) to confine the reagents. Endogenous peroxidase blocking has started by incubating the slides with hydrogen peroxide (H_2_O_2_) for 30 min at room temperature (RT), followed by three washes with 1X PBS. A subsequent protein-blocking step was performed for 1 h at RT.

Primary antibodies were diluted in 1% bovine serum albumin (BSA) prepared in 1X PBS and applied to the tissue sections, which were then incubated at 4 °C in the dark for 24 h. After incubation, slides were washed three times with 1X PBS at RT. The biotinylated secondary antibody (Goat Anti-Polyvalent) was applied for 30 min, followed by the streptavidin–peroxidase enhancer for 20 min, with intervening washes in 1X PBS.

Chromogenic detection was performed by a pathologist using a 1:50 dilution of 3,3′-Diaminobenzidine (DAB), with staining development monitored under a microscope for 1–2 min. After DAB development, the slides were rinsed in distilled water, counterstained with hematoxylin for 30 s, and then washed in distilled water.

### 2.8. Sections Imaging

Images of the stained sections were acquired using an Olympus BX63 microscope equipped with an Olympus DP80 camera (resolution of 5760 × 3600 pixels and a pixel size of 5.86 × 5.86 mm, Olympus, Tokyo, Japan) and Cell Sens Dimension 2.3 software (Build 18987). Images were acquired with 20x (numerical aperture 0.45; 1920 × 1200 pixels; 465.079 nm/pixel resolution in both X and Y axes) and 40x (numerical aperture 0.6; 1920 × 1200 pixels; 232.54 nm/pixel resolution in both X and Y axes) objective lenses.

### 2.9. Expression Evaluation

For immunohistochemical scoring, a semiquantitative system based on staining intensity was used. The tissues were classified into four categories. A score of 0 indicates undetectable immunohistochemical staining. A score of 1 indicates weak immunoreactivity/tissue expression, a score of 2 indicates moderate staining, and a score of 3 indicates strong expression. All the slides were scored by two independent observers and reviewed by a histopathologist to reach consensus. AOIs that contained no artifacts were used as a single AOI per animal in the estimation process. Section processing was automated, but expression estimation was performed manually. Because consensus scoring rather than independent final scoring was used, formal interobserver agreement statistics (e.g., weighted kappa) were not calculated. The observers and the histopathologist were blinded to the groups by using a code.

### 2.10. Statistical Analyses

Comparisons were performed using a *t*-test to compare the two groups at different time points (for the weight as an initial comparison), further testing to detect differences in weight gain was performed using a linear mixed-effects model with treatment, week, treatment × week interaction as fixed effects and mouse as a random intercept, diagnostic checks indicated homogeneous within-mouse residual variance between groups, but heterogeneous between-mouse (random-intercept) variance, which is disclosed as a limitation; a sensitivity analysis allowing group-specific random-intercept variances did not materially change the interaction estimate.) Exact Mann–Whitney U tests followed by Benjamini–Hochberg false-discovery-rate (BH-FDR) correction were performed to account for multiple testing across the seven markers evaluated in four organs (semiquantitative evaluation of 7 markers: CD3, CD8a, CD68, CD278, LPS, LTA, TGM2 × 4 organs: intestine, liver, lung, kidney). All data related to the later statistics are summarized in Supplementary Table S3. A *p*-value below 0.05 was considered significant for group differences. The study was not powered for confirmatory inference, and the estimates are preliminary because the groups were small and unbalanced. Blinding was not followed during the statistical analyses.

## 3. Results

**Figure 1:**
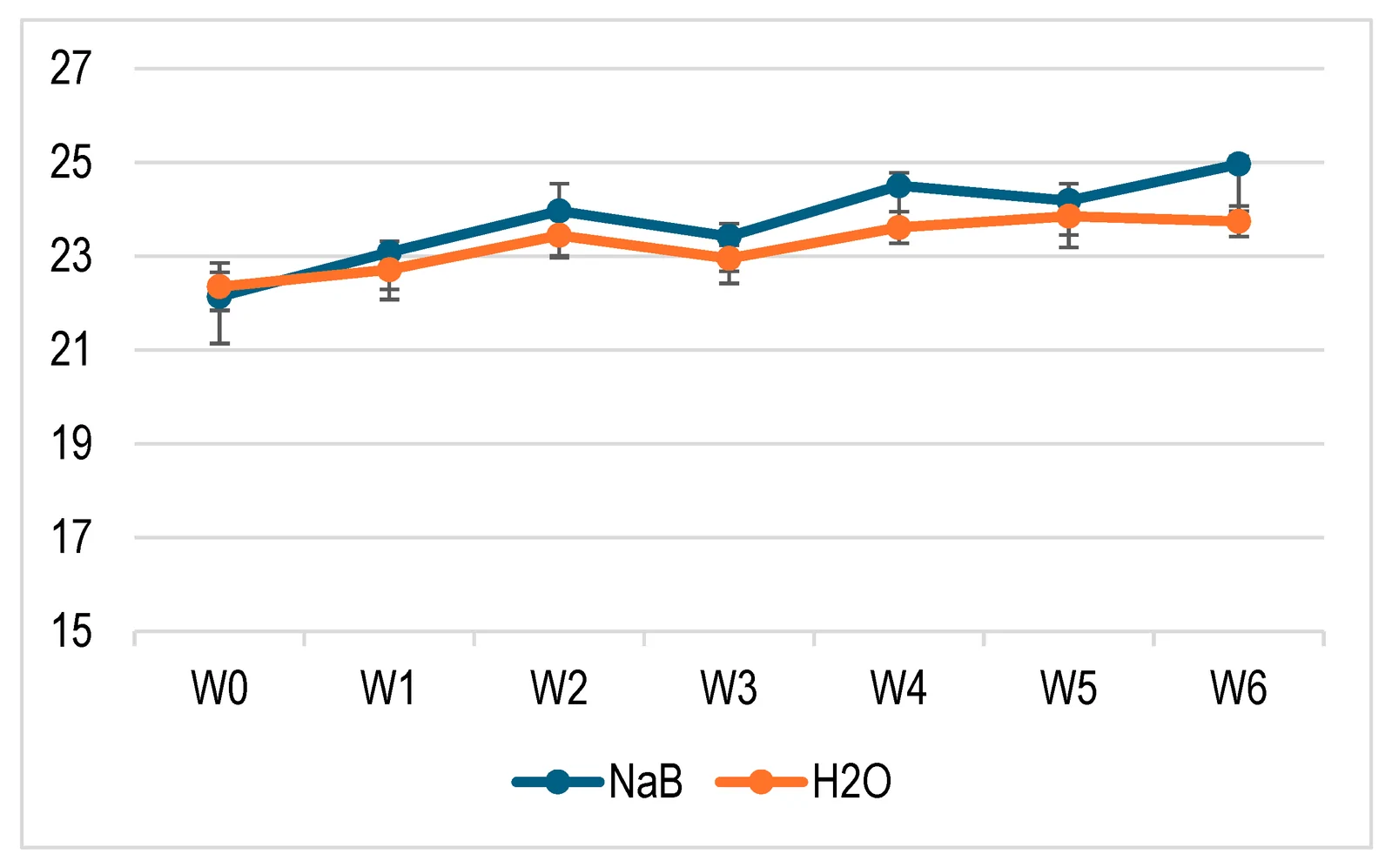
Weight follow-up during the study duration (Week 1 to Week 6). Weight is presented in grams (Y-axis). Points represent the mean weight of the mice in the group, and error bars represent the SD of the mean. NaB = 5 mice, H_2_O = 10 mice.

At the end of the study, sodium butyrate-fed mice (*n* = 5) showed increased weight compared with the water-only group (*n* = 10), and both groups remained similar in activity and well-being throughout the study (Figure 1). Body weight was analyzed longitudinally because repeated weekly measurements from the same animal are correlated (interclass correlation coefficient, ICC = 0.61). Baseline weight did not differ between groups (treatment effect at week 0: β = 0.02 g, 95% CI −0.90 to 0.94, *p* = 0.96). However, NaB-treated mice gained weight at a significantly faster rate than H_2_O controls (treatment × week interaction: β = 0.164 g/week, 95% CI 0.017–0.311, *p* = 0.028), corresponding to slopes of 0.400 g/week (NaB) versus 0.236 g/week (H_2_O). Given the small, unbalanced sample size (NaB *n* = 5, H_2_O *n* = 10), this finding should be interpreted as a preliminary, hypothesis-generating signal rather than confirmatory evidence of a NaB-induced weight-gain effect. Refer to Supplementary Tables S1 and S2 for further details.

**Figure 2:**
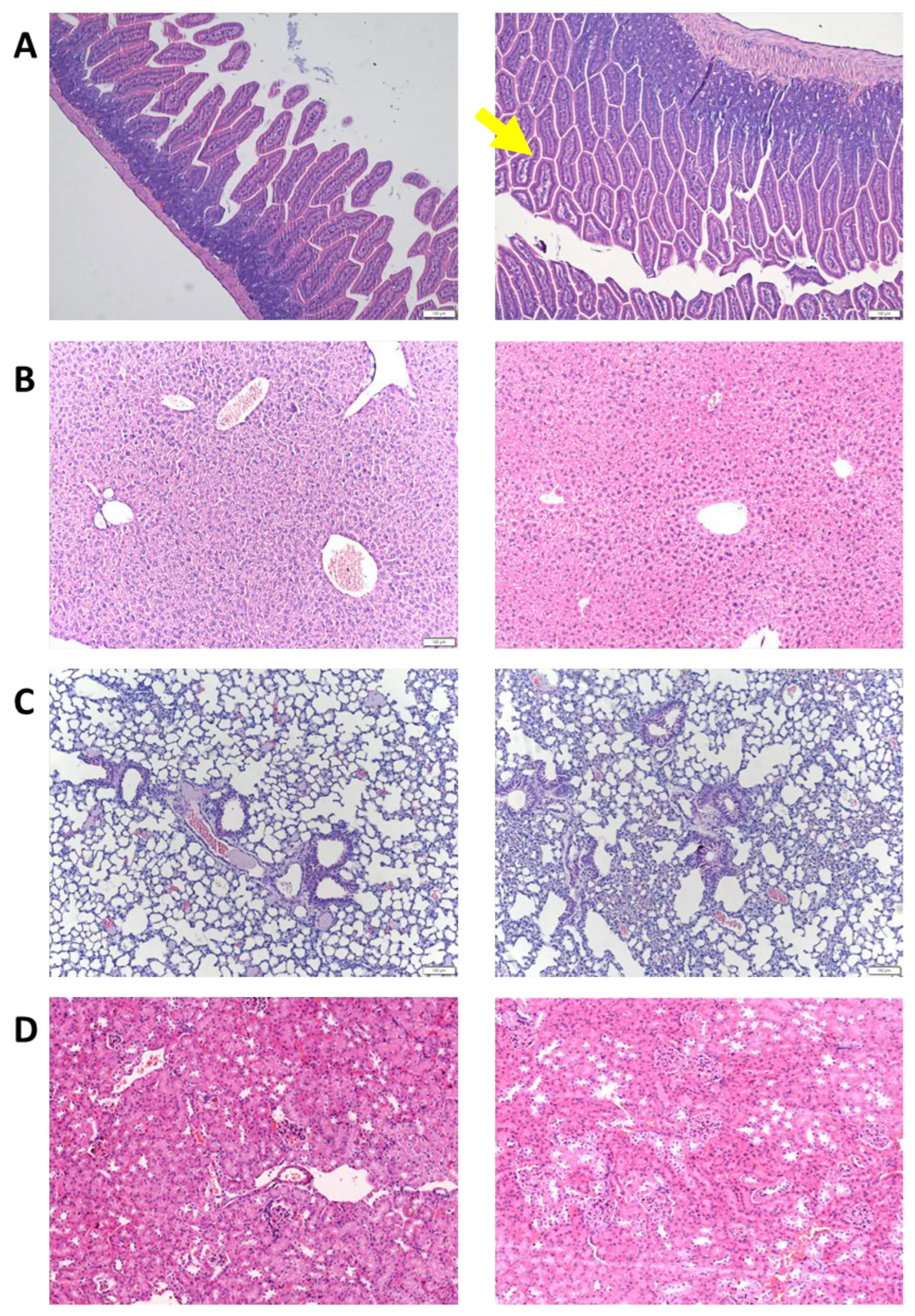
Microscopic examination of an organ using H & E staining: H_2_O-group on the left and NaB group on the right. (**A**) Small Intestine, (**B**) Liver, (**C**) Lung, (**D**) Kidney. 200×, scale bar represents 100 microns (equal in all images), note the condensed epithelial lining of the intestine in the NaB group (yellow arrow).

There were no visible differences in the gross morphology of the organs collected between the two groups. Similarly, microscopic examination with a Hematoxylin and Eosin stain showed no differences, except for condensed crypts in the small intestine of the NaB group (Figure 2).

**Figure 3:**
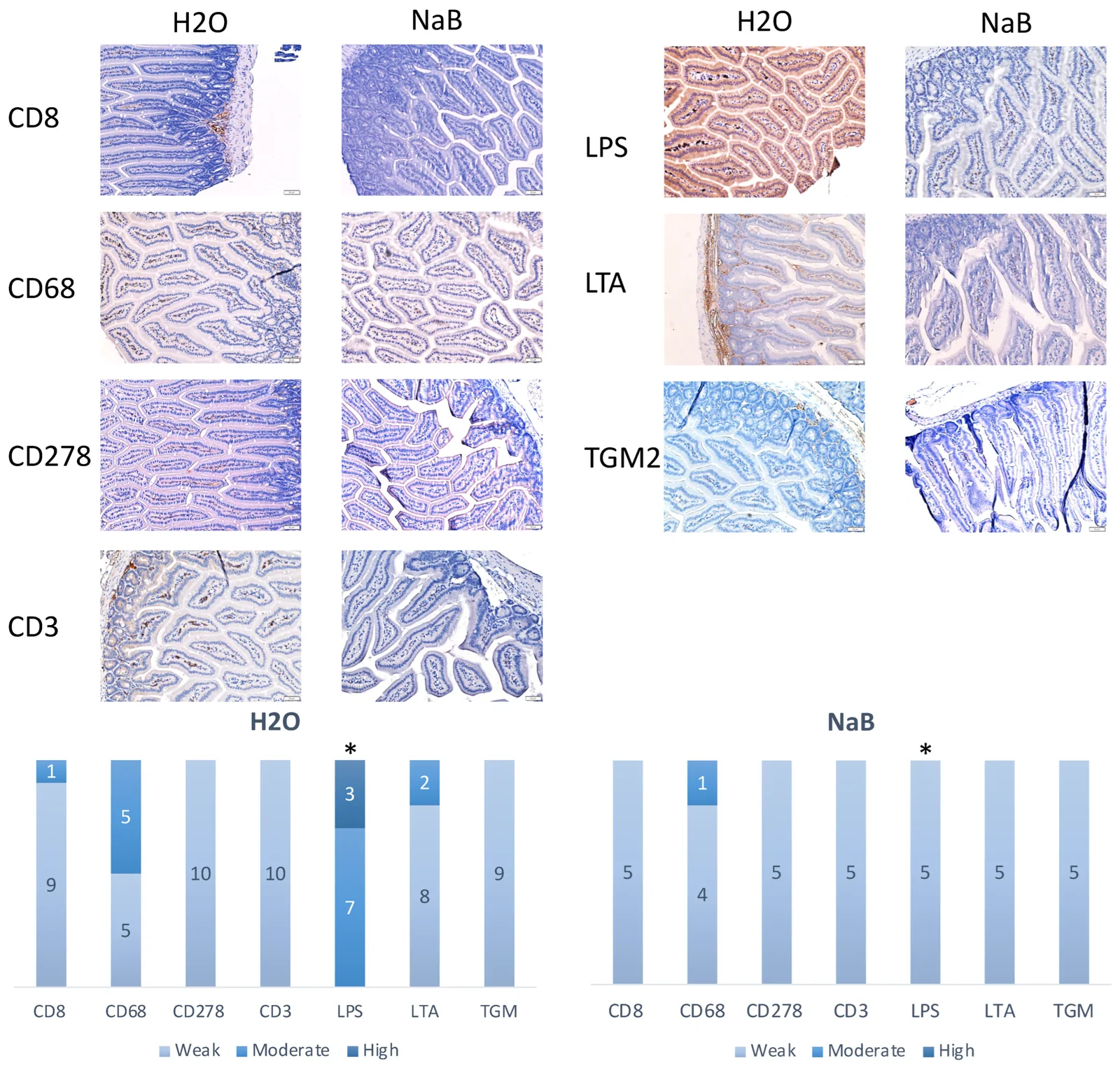
Immunohistochemical assessment of the intestinal mucosa in the regular water-fed (H_2_O) and Sodium butyrate-fed (NaB) groups showing different immune cell markers, lipopolysaccharides (LPS), Lipoteichoic acid (LTA), and Tissue Transglutaminase 2 (TGM2). Bottom left and right: graphical representation of antibody expression (weak, moderate, high) as the number of animals per group (H_2_O, bottom left, and NaB, bottom right). Scale bar: 50 microns. * Indicates a significant difference in LPS expression between NaB and H_2_O using the Exact Mann–Whitney U test with BH-FDR correction (*p* < 0.05).

Despite no significant statistical differences being detected, the intestinal immunohistochemical assessment shows an overall low basal immune signal, with most markers recorded as weak, suggesting limited immune cell infiltration or activation under the tested conditions (Figure 3). As expected in this mouse genotype, CD3 and CD8 are predominantly weak throughout, suggesting minimal T-cell presence or activation in the intestinal tissue in both groups. All other markers, including CD68, CD278, LTA, and TGM, were uniformly weak, with no group differences, as determined by the Exact Mann–Whitney U test with correction. In contrast, LPS was significantly expressed in the intestine of the H_2_O group (moderate to weak), whereas all samples showed weak detection in the NaB group (exact *p* = 0.0016, Mann–Whitney U test, adjusted *p* = 0.0112 with BH-FDR correction; Figure 3; Supplementary Table S3).

**Figure 4:**
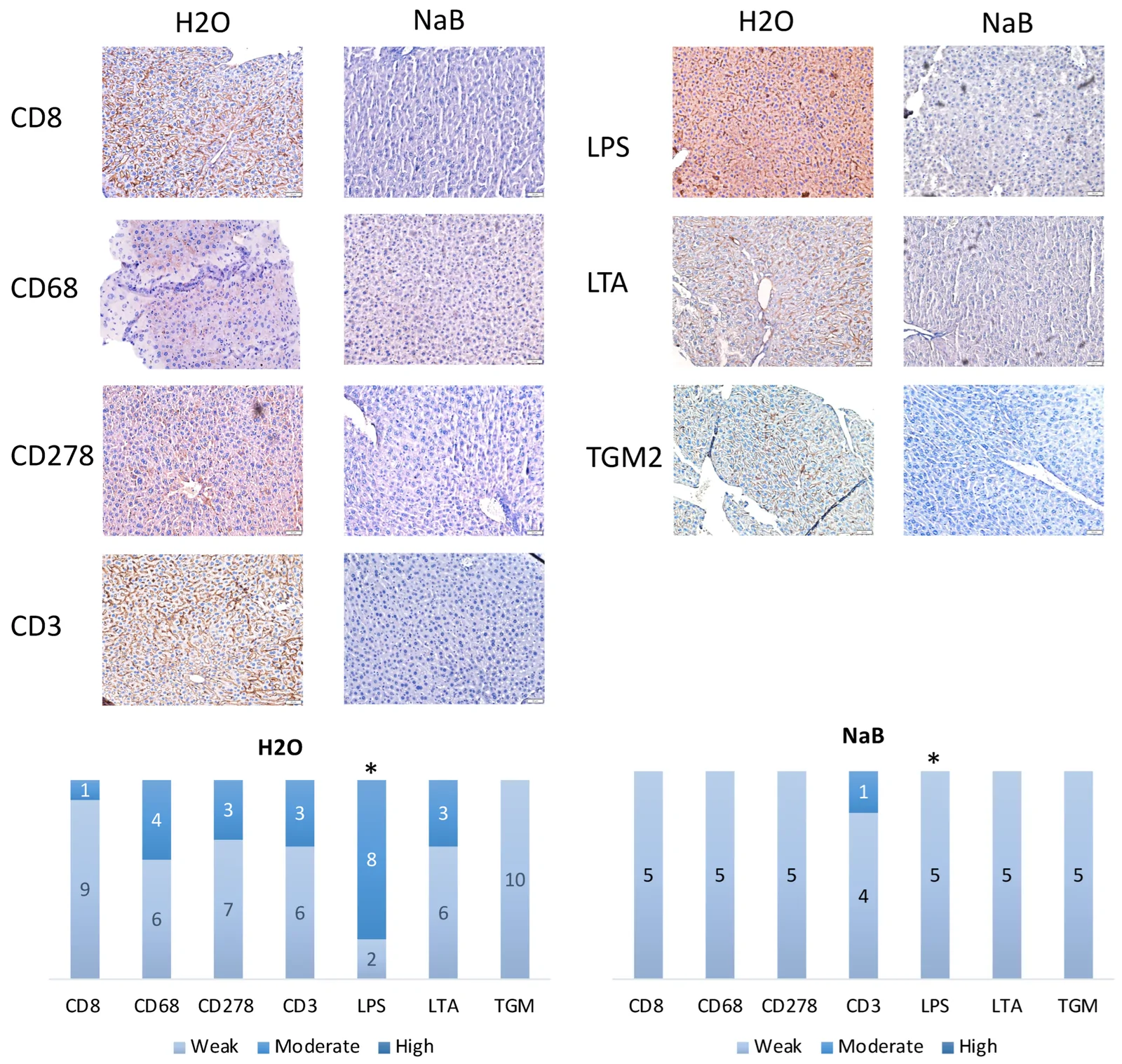
Immunohistochemical assessment of the Liver in the regular water-fed (H_2_O) and Sodium butyrate-fed (NaB) groups showing different immune cell markers, lipopolysaccharides (LPS), Lipoteichoic acid (LTA), and Tissue Transglutaminase 2 (TGM2). Bottom left and right: graphical representation of the antibody expression (weak, moderate, high) as the number of animals per group (H_2_O, bottom left, and NaB, bottom right). Scale bar: 50 microns. * Indicates a significant difference in LPS expression between NaB and H_2_O using the Exact Mann–Whitney U test with BH-FDR correction (*p* < 0.05).

Following the portal circulation flow, the liver is discussed next (Figure 4). When the intestinal findings are considered alongside the liver results, a coordinated gut–liver immune relationship is suggested. As observed in the intestines, a restrained baseline is present in the liver across most markers, with mostly weak signals for CD8, CD68, CD278, CD3, LPS, LTA, and TGM, especially in the NaB group, while LPS is also significantly expressed in the H_2_O group (exact *p* = 0.0014, Mann–Whitney U test, adjusted *p* = 0.0098 with BH-FDR correction; Figure 4; Supplementary Table S3). This parallels the proposal that controlled intestinal immune activation may be reflected in the liver via the portal circulation. However, statistics did not show a significant difference in immune cell expression between the groups.

**Figure 5:**
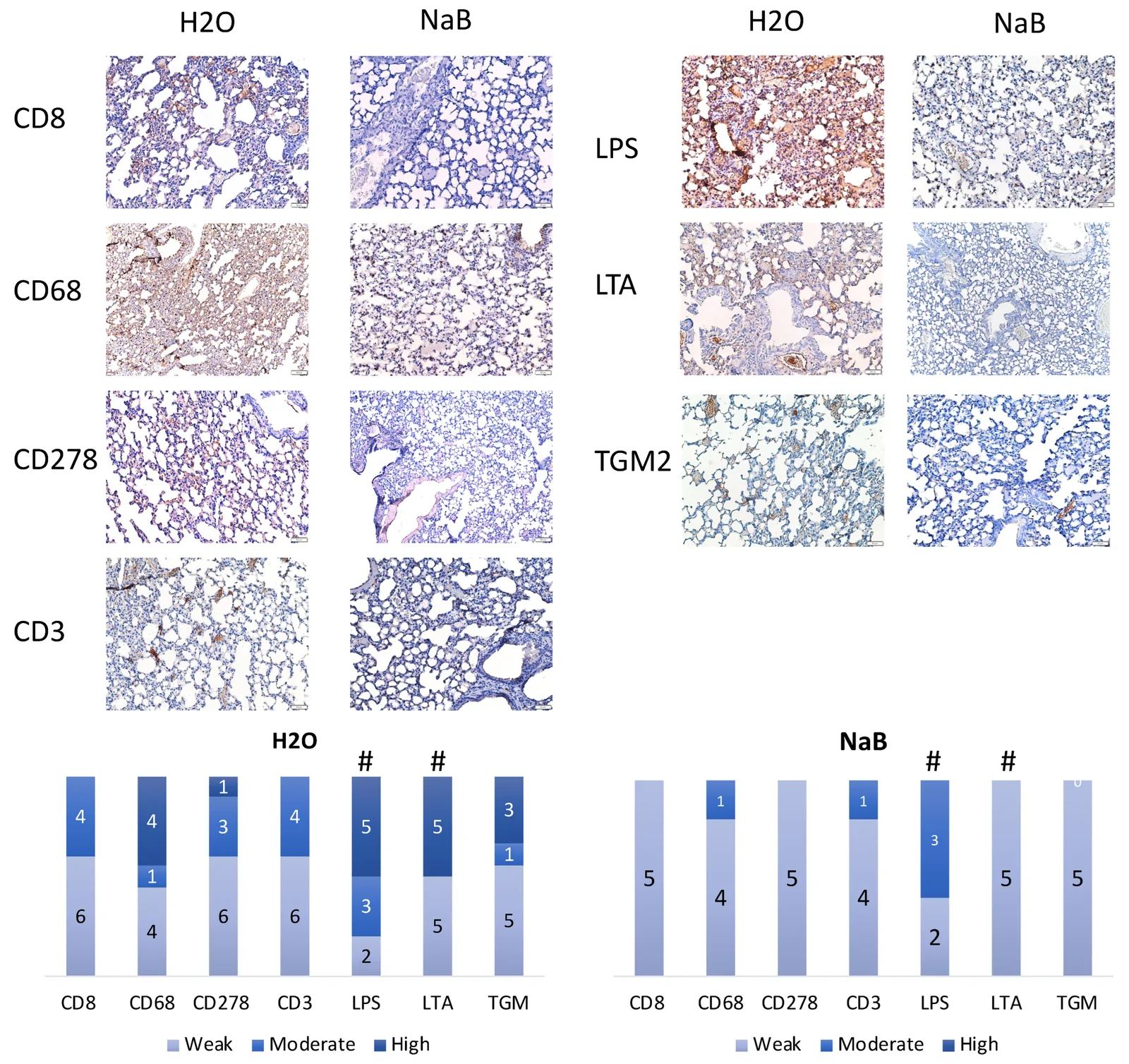
Immunohistochemical assessment of the Lung in the regular water-fed (H_2_O) and Sodium butyrate-fed (NaB) groups showing different immune cell markers, lipopolysaccharides (LPS), Lipoteichoic acid (LTA), and Tissue Transglutaminase 2 (TGM2). Bottom left and right: graphic representation of the antibody expression (weak, moderate, high) as the total number of animals per group (H_2_O, bottom left, and NaB, bottom right) Scale bar: 50 microns. # Indicates a borderline significant difference in LPS expression between NaB and H_2_O using the Exact Mann–Whitney U test with BH-FDR correction (*p* = 0.049).

The lung samples exhibit a more heterogeneous immune profile than the intestine, particularly in the H_2_O group (Figure 5). CD8, CD3, CD278, and TMG do not show significant differences between the groups (Supplementary Table S3). CD68 is significantly elevated in H_2_O samples (*p* = 0.03 according to the Exact Mann–Whitney U test), which might reflect more presence of macrophages. However, this difference did not survive the correction for multiple comparisons (and we report it here to show a trend). Similarly, bacterial antigen levels (LPS and LTA) are elevated in the H_2_O group (*p* = 0.021 and 0.014 in the Exact Mann–Whitney U test, respectively), but with correction the two markers can only be labeled as borderline (*p* = 0.049), which is directionally supported by the raw ordinal data (NaB skewed toward weak/moderate while H_2_O skewed toward moderate/high). Collectively, the lung data show a trend toward more bacterial antigenic and innate immune expression in the H_2_O group compared with the NaB group (Figure 5).

**Figure 6:**
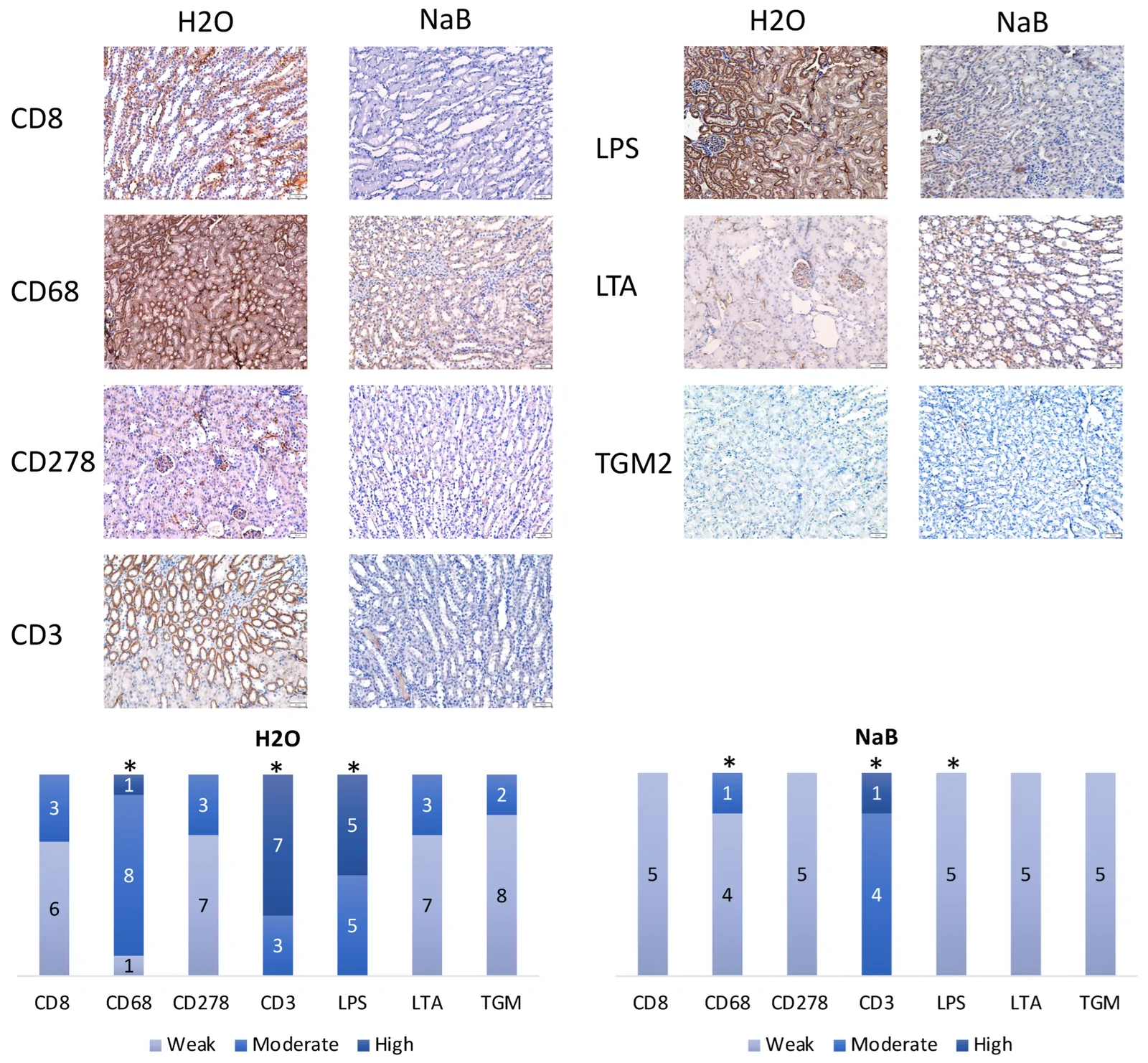
Immunohistochemical assessment of the Kidney in the regular water-fed (H_2_O) and Sodium butyrate-fed (NaB) groups showing different immune cell markers, lipopolysaccharides (LPS), Lipoteichoic acid (LTA), and Tissue Transglutaminase 2 (TGM2). Bottom left and right: graphical representation of the antibody expression (weak, moderate, high) as a percentage of the total number of animals per group (H_2_O, bottom left, and NaB, bottom right). Scale bar: 50 microns. * Indicates a significant difference in LPS expression between NaB and H_2_O using the Exact Mann–Whitney U test with BH-FDR correction (*p* < 0.05).

The kidney exhibits a distinct immune profile, with CD3 generally low in the NaB group but increasing to moderate or high levels in several H_2_O samples (exact *p* = 0.0089; Mann–Whitney U test; adjusted *p* = 0.021 with BH-FDR correction). CD68 also shows more expression in H_2_O samples, indicating notable macrophage activity, whereas it remains significantly weaker in NaB-treated samples (exact *p* = 0.0027; Mann–Whitney U test; adjusted *p* = 0.0095 with BH-FDR correction). LPS responses are consistently and significantly elevated in the H_2_O group (exact *p* = 0.0003; Mann–Whitney U test; adjusted *p* = 0.0027 with BH-FDR correction). No significant differences are seen for the other markers. (Figure 6, Supplementary Table S3).

## 4. Discussion

This study examined the systemic and organ-specific bacterial antigenic and immune profiles of sodium butyrate (NaB) supplementation in nude athymic mice, focusing on gut–liver–lung–kidney immune interactions under conditions of limited adaptive immunity. To our knowledge, this is the first study to demonstrate the immunological effects of butyrate in an athymic, immunodeficient mouse model. Overall, the results indicate that NaB has potential immunomodulatory effects, reducing microbial antigen detection and decreasing innate immune expression across multiple organs, without causing obvious toxicity or tissue damage. Specifically, NaB supplementation was associated with lower LPS expression in the small intestine, liver, and kidney, with a borderline decrease in the lungs. The immune profile also showed a reduction in the macrophage marker in the kidneys.

Body weight and general well-being were comparable between groups, although NaB-treated mice gained higher average body weight per week. A review report by Zhang et al. found a weight-reducing or control effect of butyrate across various rodent models, while some researchers reported no change [18]. Short-chain fatty acids such as butyrate can modestly enhance metabolic efficiency and intestinal energy harvest, which may partially explain this observation [2]. Importantly, the absence of behavioral or morphological abnormalities suggests that NaB was well tolerated in our immunodeficient model.

Histological analysis revealed largely preserved tissue architecture across all organs, except for condensed intestinal crypts in NaB-treated mice. Butyrate can influence epithelial turnover and differentiation by acting as a histone deacetylase inhibitor, thereby altering crypt morphology without necessarily inducing inflammation [3,19]. Thus, the crypt condensation observed here may reflect epithelial maturation rather than pathology.

Immunohistochemical analysis of the intestine showed an overall weak basal immune signal, consistent with the nude athymic genotype. As expected, CD3 and CD8 expression was predominantly weak in both groups. An earlier report demonstrated that nude mice, while lacking a functional thymus, can retain small populations of extrathymically derived T-like cells, which may account for weak CD3 or CD8 staining [20]. In addition, antibody cross-reactivity or expression of shared epitopes by innate lymphoid cells may further contribute to detectable signals [20,21]. Therefore, the presence of weak T-cell markers in this model does not necessarily contradict the athymic phenotype, yet it should be interpreted with caution, as non-specific binding can be a contributing factor [22]. The presence of weak expression in both groups, with no significant difference, despite some scattered moderate expressions, cannot therefore be interpreted as a treatment-dependent finding.

Among microbial antigens, LPS showed the strongest organ-specific detection, particularly in the intestine, liver and kidney of the H_2_O group. In a different but related context, Szentirmai et al. found that LPS can be detected in the systemic circulation even under homeostatic conditions due to continuous microbial sampling [23]. Notably, NaB supplementation was markedly associated with lower LPS detection, which is potentially consistent with, but does not directly demonstrate, improved barrier function. This aligns with findings by Bakshi et al. and others, who reported that butyrate strengthens tight junctions and suppresses endotoxin penetration [3,24]. We could not prove statistically that innate immune markers (namely CD68) were affected by NaB supplementation in association with the LPS profile, except in the kidney. A study by Liang et al. suggested that butyrate promotes anti-inflammatory macrophage phenotypes, which may increase marker expression without inducing inflammation; however, this was illustrated only in the kidney of our mice [25].

The liver findings mirror those of the intestine, so parallel, statistically independent reductions in LPS immunoreactivity in both the intestine and the liver were observed. While the current study design cannot be used to reinforce the concept of a coordinated gut–liver axis via the portal circulation, this remains a plausible biologic interpretation. Studies in various animal models and across different LPS administration routes have demonstrated that intestinal barrier dysfunction directly amplifies hepatic inflammation, and that butyrate helps reduce LPS-induced inflammation in the gut-liver axis [25,26,27].

The lung exhibited a borderline elevation of LPS and LTA in the H_2_O group, which might suggest heightened sensitivity to inhaled microbial components, housing exposure, circulating antigens, or nonspecific staining, rather than originating from the gut. Smith et al. found that systemic endotoxemia can prime pulmonary macrophages, even in the absence of overt infection [28], and this can be correlated with the observed expression of LPS and LTA markers in the lung and the ordinal increase in CD68 in the lung tissue, despite not surviving the correction. Intestinal modulation can exert distal anti-inflammatory effects, supporting the concept of a gut–lung axis as illustrated in other animal models [9,10,11], which is likely replicable in our model with a design targeted to demonstrate the involvement of this axis. Similarly, the kidney showed intermediate immune activation, with increased expression of CD3, CD68, and LPS in the H_2_O group, a pattern not observed in NaB-treated mice. The reduced expression of these markers in NaB-treated mice suggests protection against systemic microbial toxins and attenuation of LPS-induced kidney inflammation, as reported previously [12,13,14].

Several limitations of the present study should be acknowledged. First, the small and unbalanced sample size (NaB, *n* = 5; H_2_O, *n* = 10) represents a primary constraint, limiting statistical power and the ability to detect subtle between-group differences with confidence. This was an inherent consequence of working within the ethical framework and the logistical demands of maintaining athymic nude mice under strict sterile conditions. Accordingly, the present study should be regarded as exploratory and preliminary, and findings should not be generalized until confirmed in larger, adequately powered cohorts with formal a priori sample size calculations. Second, the semiquantitative IHC scoring system introduces an element of subjectivity, while nonparametric analyses of the semi-quantifications were applied to ensure statistical soundness. Third, the assessment of intestinal permeability was not performed using functional assays or by measuring tight junction protein expression (e.g., ZO-1, occludin), at the mRNA or protein level, nor were circulating LPS levels or cytokine profiles quantified, as these were beyond the scope of the study. Yet, these measures represent necessary future directions of this study. As a result, the proposed mechanisms linking NaB treatment to reduced microbial detection remain associative rather than causally established. Fourth, microbiome composition was not assessed, limiting the ability to attribute observed changes in microbial antigen staining to specific shifts in the gut microbial community. Finally, although body weight was monitored weekly and, given the lack of confirmation of NaB stability in the study, we cannot rule out the possibility that the strong odor and taste of sodium butyrate may have influenced voluntary water consumption. Future studies should incorporate larger, more balanced cohorts or alternative immunocompromised models, functional barrier integrity assays, molecular markers of tight junction proteins, microbiome profiling, and circulating inflammatory biomarkers to validate and extend the preliminary findings reported here.

In summary, this study showed that NaB supplementation was associated with reduced detection of tissue-associated bacterial antigens and altered immune marker expression, with the possibility to regulate the innate immune environment across multiple organs in nude athymic mice. The lower detection of microbial antigens, particularly LPS, appears central to this effect. Together, these findings highlight the capacity of dietary metabolites to modulate innate immune pathways, even in severely immunocompromised models, with implications for multi-organ systemic immune regulation.

## Figures and Tables

**Table 1:** Descriptions of the used antibodies.

Antibody	Optimized Dilution	Optimized Ag Retrieval (pH)	Catalogue No.	Description
CD3	1:200	pH 9.0	344802	Cytoplasm T cell
LPS	1:200	pH 9.0	Ab35654	Gram (−ve)
CD8a	1:200	pH 6.0	372902	Membrane/Nucleus (Killer T cells)
CD278	1:1000	pH 6.0	A1811	Membrane (Immune checkpoint)
TGM2	1:600	pH 6.0	Ab2386	Cytoplasm/cell surface (adhesion)
LTA	1:100	pH 6.0	NBP1-60146-0.1 mg	Gram (+ve)
CD68	1:1000	pH 9.0	Ab303565	Macrophage Marker

## Data Availability

The original contributions presented in this study are included in the article and Supplementary Materials. Further inquiries can be directed to the corresponding author.

## Supplementary Materials

The following supporting information can be downloaded at: https://www.mdpi.com/article/10.3390/nu18162604/s1, Table S1: Body weight comparisons for the two groups. Table S2: Fixed-effect estimates of Weight Change. Table S3: Comparisons between different marker expressions in the organs tested using Exact Mann-Whitney U test and the Benjamini–Hochberg false-discovery-rate correction. Table S4: Figures of the positive controls.
