# Supplementary material for: Oral Sodium Butyrate Is Associated with Altered Tissue Bacterial Antigens and Innate Immune Marker Expression Across Different Organs in Athymic Mice
Source: Nutrients. 2026 Aug 10;18(16):2604. doi: 10.3390/nu18162604 (PMC13516814; doi:10.3390/nu18162604)
Supplement: Supplementary file 1 [file nutrients-18-02604-s001.zip › nutrients-4388876-supplementary.pdf]

Supplementary Table S1: Body weight comparisons for the two groups

**Week**	**NaB Mean (±SD)**	**H2O Mean (±SD)**	**p-value (t-test)**
**1**	19.38 (±1.68)	20.36 (±2.84)	0.2536
**1**	22.60 (±1.79)	21.48 (±2.21)	1.0000
**2**	22.88 (±1.32)	22.22 (±1.87)	0.2536
**3**	22.14 (±1.28)	22.25 (±1.56)	0.2536
**4**	23.08 (±0.59)	22.51 (±1.17)	0.2536
**5**	23.96 (±1.19)	23.09 (±1.38)	0.3535
**6**	23.42 (±0.59)	22.95 (±0.95)	0.2536

Supplementary Table S2: Fixed-effect estimates of Weight Change

Parameter*	Estimate	SE	95% CI	t (df=90)	p-value
**Intercept (H2O, Week 0)**	22.53 g	0.29	21.95 – 23.11	—	—
**Treatment (NaB vs. H2O), baseline**	+0.02 g	0.46	−0.90 – 0.94	0.05	0.96
**Week (H2O slope)**	0.236 g/wk	0.043	0.152 – 0.321	5.53	<0.001
Treatment × Week (interaction)	**+0.164 g/wk**	**0.074**	**0.017 – 0.311**	**2.22**	**0.028**

*Group-specific slopes derived from the model: H2O weekly weight gain: 0.236 g/week (95% CI 0.152–0.321), NaB weekly weight gain: 0.400 g/week (95% CI 0.280–0.520)

Random-Effects (Variance Component) Estimates

Component	Estimate	SD
**Mouse random intercept variance (τ^2, pooled)**	0.799	0.894 g
**Residual (within-mouse) variance (σ^2)**	0.509	0.713 g
**Intraclass correlation (ICC)**	0.61	—

Supplementary Table S3: Comparisons between different marker expressions in the organs tested using Exact Mann-Whitney U test and the Benjamini–Hochberg false-discovery-rate correction

Organ	Marker	Group	n	Median (IQR)	Counts (weak, mod/strong)	U statistic	Exact p-value	Adjusted p (BH)
**Intestine**	CD8	Na.B	5	1 (1–1)	w,w,w,w,w	22.5	1.00	1.00
		H2O	10	1 (1–1)	w,w,w,m,w,w,w,w,w,w			
	CD68	Na.B	5	1 (1–1)	w,w,w,w,m	15.0	0.19	0.665
		H2O	10	1.5 (1–2)	w,w,w,m,m,w,m,m,m,w			
	CD278	Na.B	5	1 (1–1)	w,w,w,w,w	25.0	1.00	1.00
		H2O	10	1 (1–1)	w×10			
	CD3	Na.B	5	1 (1–1)	w,w,w,w,w	25.0	1.00	1.00
		H2O	10	1 (1–1)	w×10			
	**LPS**	**Na.B**	**5**	**1 (1–1)**	w,w,w,w,w	**2.0**	**0.0016**	**0.0112**

		**H2O**	**10**	**2 (2–2.75)**	m,s,s,s,m,m,m,m,m,m			
	LTA	Na.B	5	1 (1–1)	w,w,w,w,w	20.0	0.53	0.99
		H2O	10	1 (1–1)	w,w,w,w,m,w,w,m,w,w			
	TGM	Na.B	5	1 (1–1)	w,w,w,w,w	22.5	1.00	1.00
		H2O#	9	1 (1–1)	w×9			
**Liver**	CD8	Na.B	5	1 (1–1)	w,w,w,w,w	22.5	1.00	1.00
		H2O	10	1 (1–1)	w,m,w,w,w,w,w,w,w,w			
	CD68	Na.B	5	1 (1–1)	w,w,w,w,w	12.5	0.089	0.208
		H2O	10	1.5 (1–2)	w,w,m,m,m,m,w,m,w,w			
	CD278	Na.B	5	1 (1–1)	w,w,w,w,w	20.0	0.53	0.53
		H2O	10	1 (1–1)	w,w,m,w,w,m,m,w,w,w			
	CD3	Na.B	5	1 (1–1)	w,w,w,m,w	17.0	0.45	0.53
		H2O#	9	1 (1–2)	w,w,m,w,w,m,w,m,w			
	**LPS**	**Na.B**	**5**	**1 (1–1)**	w,w,w,w,w	**5.0**	**0.0014**	**0.0098**
		**H2O**	**10**	**2 (1.25–2)**	w,w,m,m,m,m,m,m,m,m			
	LTA	Na.B	5	1 (1–1)	w,w,w,w,w	17.0	0.45	0.53
		H2O#	9	1 (1–2)	w,w,m,w,w,m,w,m,w			
	TGM	Na.B	5	1 (1–1)	w,w,w,w,w	25.0	1.00	1.00
		H2O	10	1 (1–1)	w×10			
**Lung**	CD8	Na.B	5	1 (1–1)	w,w,w,w,w	15.0	0.19	0.44
		H2O	10	1 (1–2)	w,w,m,w,w,m,m,m,w,w			
	CD68	Na.B	5	1 (1–1)	w,w,w,m,w	8.0	0.030	0.070
		H2O#	9	3 (1–3)	w,s,s,s,m,w,s,w,w			
	CD278	Na.B	5	1 (1–1)	w,w,w,w,w	17.5	0.34	0.60
		H2O	10	1 (1–2)	w,w,s,w,w,m,m,m,w,w			
	CD3	Na.B	5	1 (1–1)	w,w,w,m,w	17.5	0.34	0.60
		H2O	10	1 (1–2)	w,w,m,w,w,m,m,m,w,w			
	LPS	Na.B	5	2 (1–2)	m,w,m,m,w	7.5	0.021	0.049*
		H2O	10	3 (2–3)	m,m,s,s,w,m,s,s,w,s			
	LTA	Na.B	5	1 (1–1)	w,w,w,w,w	6.5	0.014	0.049*
		H2O	10	2.5 (1–3)	w,w,s,w,w,s,s,s,s,m			
	TGM	Na.B	5	1 (1–1)	w,w,w,w,w	15.0	0.28	0.60
		H2O#	9	1 (1–3)	w,m,s,s,w,s,w,w,w			
**Kidney**	CD8	Na.B	5	1 (1–1)	w,w,w,w,w	15.0	0.13	0.23
		H2O#	9	1 (1–2)	w,w,m,w,w,m,w,m,w			
	**CD68**	**Na.B**	**5**	**1 (1–1)**	w,w,w,m,w	**3.5**	**0.0027**	**0.0095**
		**H2O**	**10**	**2 (2–2)**	m,s,m,m,m,m,m,m,m,w			

	CD278	Na.B	5	1 (1–1)	w,w,w,w,w	17.5	0.24	0.28
		H2O	10	1 (1–2)	w,w,m,w,w,m,w,m,w,w			
	**CD3**	**Na.B**	**5**	**2 (2–2)**	m,s,m,m,m	**5.0**	**0.0089**	**0.0208**
		**H2O**	**10**	**3 (2.25–3)**	s,s,s,m,m,s,m,s,s,s			
	**LPS**	**Na.B**	**5**	**1 (1–1)**	w,w,w,w,w	**0.0**	**0.00039**	**0.0027**
		**H2O**	**10**	**2.5 (2–3)**	m,s,s,s,m,m,s,s,m,m			
	LTA	Na.B	5	1 (1–1)	w,w,w,w,w	17.5	0.24	0.28
		H2O	10	1 (1–2)	w,w,w,m,w,m,w,w,m,w			
	TGM	Na.B	5	1 (1–1)	w,w,w,w,w	20.0	0.41	0.41
		H2O	10	1 (1–1)	w,w,m,w,m,w,w,w,w,w			

*Lung LPS and LTA are borderline at the BH critical boundary (adjusted p ≈ 0.049) and are reported as suggestive/non-confirmatory given the small, unbalanced sample size, rather than as confirmed significant findings.

[#] missing values in the scoring sheet due to low-quality images and incomplete tissue sections

Supplementary Table S4: figures of the positive controls:

Marker	Tissue	Image
CD68	Tonsil	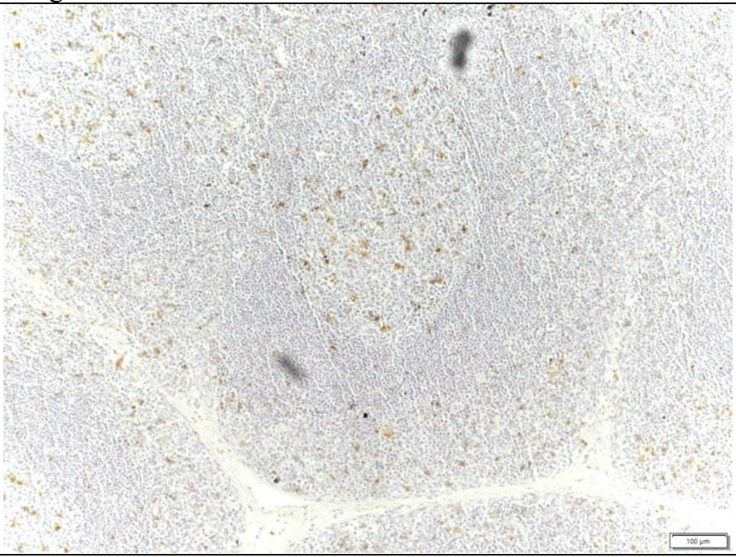

CD278	Tonsil	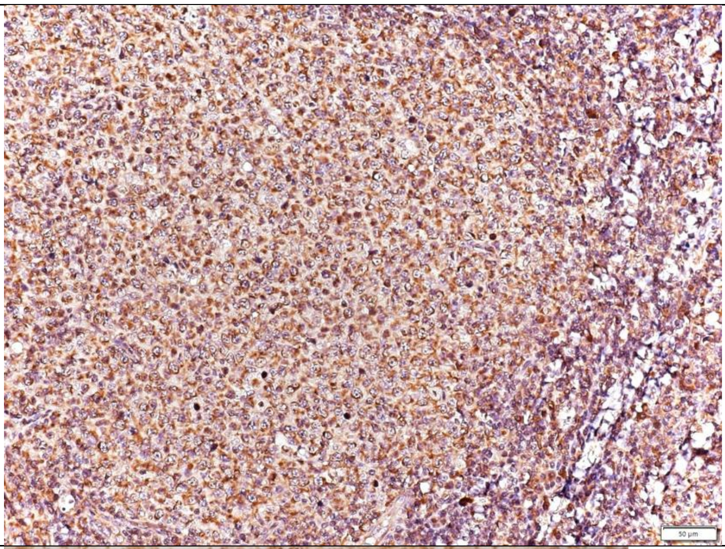
LTA	Gram positive bacteria	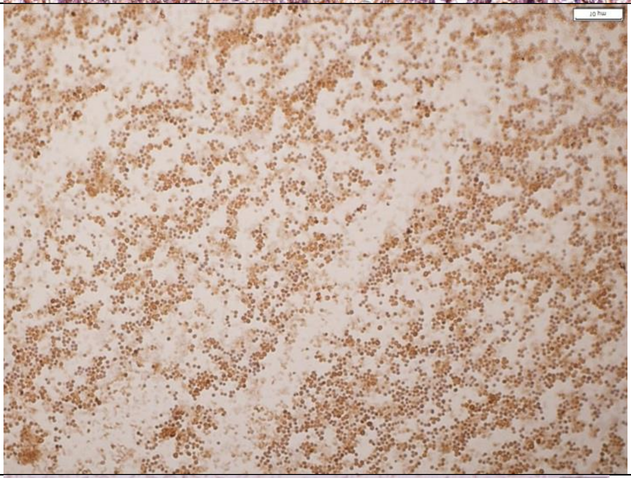
LPS	Gram negative bacteria	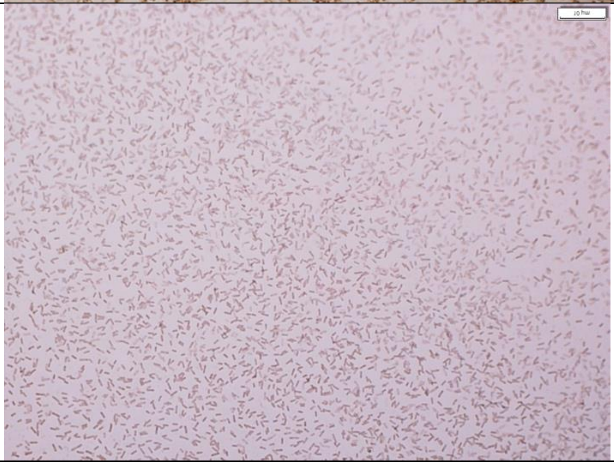

CD8	Tonsil	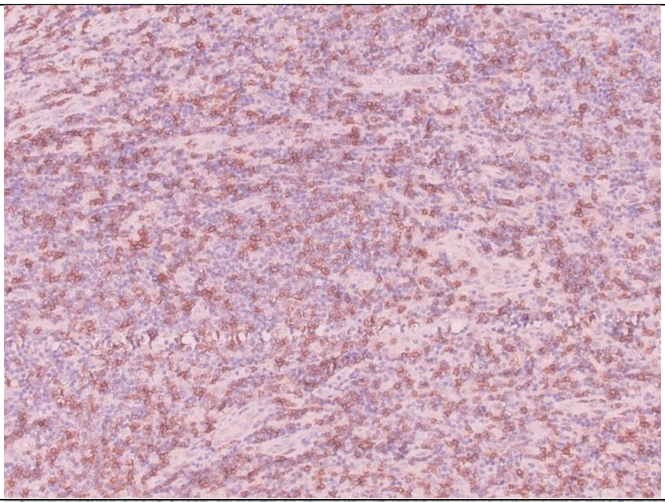
CD3	Tonsil	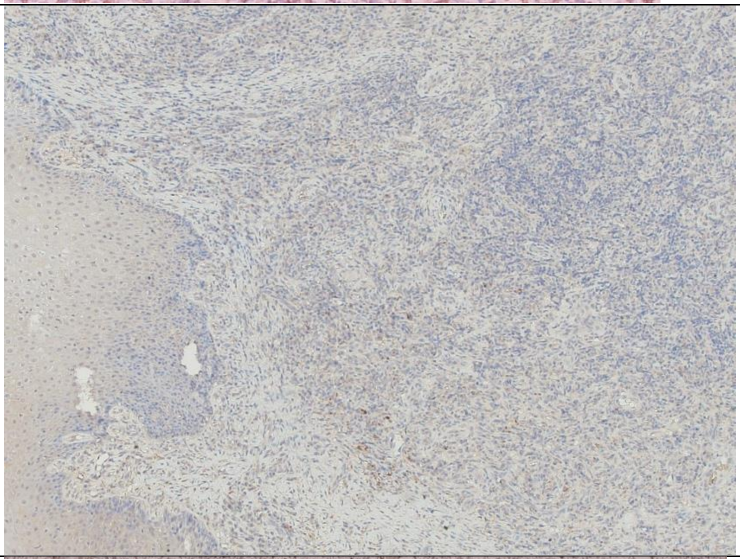
TGM2	Tonsil	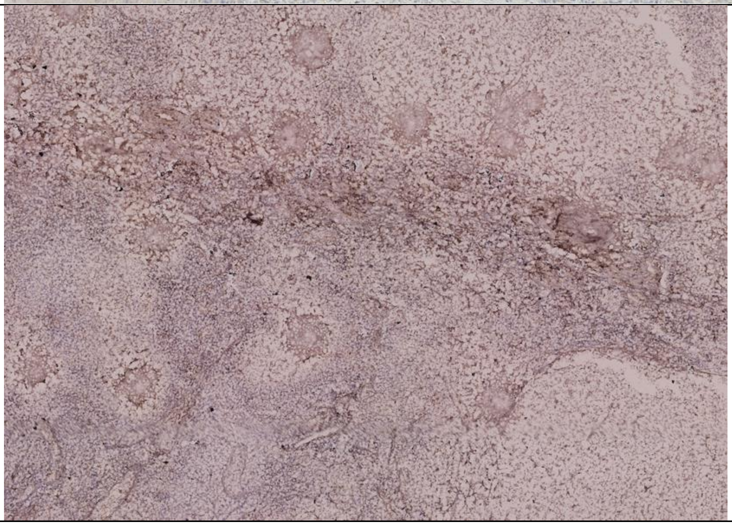
